# Silymarin modulates catabolic cytokine expression through *Sirt1* and *SOX9* in human articular chondrocytes

**DOI:** 10.1186/s13018-021-02305-9

**Published:** 2021-02-20

**Authors:** Wen-Tien Wu, Yi-Ru Chen, Dai-Hua Lu, Fedor Svyatoslavovich Senatov, Kai-Chiang Yang, Chen-Chie Wang

**Affiliations:** 1 Department of Orthopedic Surgery, Taipei Tzu Chi Hospital, Buddhist Tzu Chi Medical Foundation, No. 289, Jianguo Rd., Xindian Dist, New Taipei City, 23142 Taiwan; 2 Department of Orthopedics, Hualien Tzu Chi Hospital, Buddhist Tzu Chi Medical Foundation, Hualien, Taiwan; 3 Department of Orthopedics, School of Medicine, Tzu Chi University, Hualien, Taiwan; 4 School of Dental Technology, College of Oral Medicine, Taipei Medical University, No. 250, Wuxing St., Xinyi Dist, Taipei, 11031 Taiwan; 5 Researcher of the Centre for Composite Materials, National University of Science and Technology MISIS, Moscow, Russia

**Keywords:** Osteoarthritis, Catabolic cytokine, Silymarin, Matrix metalloproteinase, Sirtuin-1

## Abstract

**Background:**

Silymarin (SMN), a polyphenolic flavonoid, is involved in multiple bioactive functions including anti-inflammation. Pretreatment with SMN demonstrated chondroprotection against tumour necrosis factor-alpha (TNF-α) stimulation in a chondrocyte cell line. However, pre- and posttreatment with phytochemicals have varying effects on osteoarthritis (OA) chondrocytes, and the therapeutic potential of SMN after catabolic cytokine stimulation is not fully elucidated.

**Methods:**

The cytotoxicity of SMN (12.5, 25, 50 and 100 μM) was evaluated in human primary chondrocytes. The chondrocytes were supplemented with SMN (25 and 50 μM) after interleukin-1beta (IL-1β) stimulation. The mRNA expression and protein production of catabolic/anabolic cytokines as well as extracellular matrix (ECM) components were evaluated.

**Results:**

High-dose SMN (100 μM) impaired the mitochondrial activity in chondrocytes, and 50 μM SMN further caused cell death in IL-1β-stimulated cells. The addition of 25 μM SMN ameliorated cell senescence; downregulated the catabolic genes of *inducible nitric oxide synthase*, *IL*-*1β*, *TNF*-*α*, *matrix metalloproteinase-3* (*MMP*-*3*), *MMP*-*9* and *MMP*-*13*; upregulated the anabolic genes of *tissue inhibitor of metalloproteinase*-*1* (*TIMP*-*1*) and *collagen type II alpha 1*; and restored the expression of chondrogenic phenotype genes *SOX9* and *sirtuin*-*1* (*Sirt1*). In addition, the production of IL-1β, MMP-3 and MMP-9 decreased with an increase in TIMP-1 secretion. However, the mRNA levels of IL-6, IL-8 and IL-10 and protein production remained high. The addition of nicotinamide, a *Sirt1* inhibitor, downregulated *SOX9* and attenuated the therapeutic effects of SMN on IL-1β-stimulated chondrocytes.

**Conclusion:**

SMN regulates the chondrocyte phenotype through *Sirt1* and *SOX9* to improve ECM homeostasis and may serve as a complementary therapy for early-stage knee OA.

## Background

Disturbances in extracellular matrix (ECM) anabolism and catabolism are triggered by metabolic inflammation and oxidative stress and are known to initiate knee osteoarthritis (OA)-related pathological changes [1]. In particular, catabolic cytokines such as matrix metalloproteinases (MMPs) contribute to the degradation of the ECM components type II collagen and aggrecan [2]. Consequently, the imbalance in ECM deposition and degradation may result in cartilage destruction and eventually impair mobility in patients with OA [3]. Accordingly, interventions to modulate ECM homeostasis as well as to enhance the anti-inflammatory and antioxidant activities of articular chondrocytes are a promising therapeutic strategy against OA in the early stages [4].

Phytochemicals, involved in various bioactive functions, may be a promising choice for OA management [5]. Silymarin (SMN), a polyphenolic flavonoid, exerts anticarcinogenic, anti-inflammatory, cytoprotective and antioxidant effects, as evident in its radical scavenging activities, by regulating various cell membrane and nuclear transporters [6]. SMN reduced hepatic collagen accumulation through downregulation of procollagen α1 in rats with secondary biliary cirrhosis [7]. SMN also exerted cardioprotective effects on rats with myocardial infarction [8]. In a rat paw oedema model, SMN also exhibited anti-inflammatory activity [9]. In addition, SMN pretreatment before tumour necrosis factor alpha (TNF-α) exposure exerted chondroprotection through the modulation of *interleukin 6* (*IL*-*6*), *IL*-*8* and *MMP*-*1* mRNA expression in human chondrocyte cells [10]. In a monoiodoacetate-induced rat OA model, SMN enhanced the anti-inflammatory activity of the nonsteroidal anti-inflammatory drug (NSAID) celecoxib [11]. Similarly, oral administration of SMN alone or in combination with an NSAID (piroxicam) reduced serum IL-1α and IL-8 levels in patients with knee OA [12]. Moreover, combination therapy with SMN reduced the adverse events of the NSAID celecoxib in an OA rat model [13].

Despite the chondroprotective potential of SMN revealed in previous studies, the benefits of pre- and posttreatment of phytochemicals for OA chondrocytes varied [14]. Furthermore, different cytokines cause glycophenotypic alterations and regulate distinctive apoptosis signalling in human articular chondrocytes [15, 16]. Therefore, this study aimed to evaluate the therapeutic effects of SMN post-treatment on human chondrocytes.

## Materials and methods

### Human articular chondrocyte isolation and cultivation

The experimental protocol and retrieval process of human tissues were reviewed and approved by the Research Ethics Committee of Hualien Tzu Chi Hospital, Buddhist Tzu Chi Medical Foundation (IRB106-48-A). Written informed consent was obtained from all patients. Articular cartilage samples were harvested from 12 patients with advanced knee OA (five women and five men; age range, 67–84 years; average age, 75.8 ± 6.2 years). An additional two women and one man (age range, 63–80 years; average age, 70.7 ± 8.6 years) were enrolled for a *Sirt1* study. The collected cartilaginous tissues were treated with 0.1% protease (P8811, Sigma-Aldrich, Saint Louis, MO, USA) for 30 min and then digested using 0.2% type II collagenase (9001-12-1, Gibco, USA) overnight for chondrocyte isolation [17]. The released cells were maintained in Dulbecco’s modified Eagle’s medium: Nutrient Mixture F-12 (10565018, Gibco) supplemented with 10% fetal bovine serum (SH30396.03, Hyclone, USA) and 1% antibiotic (15140-122, Gibco) in an incubator at 37 °C in a humidified atmosphere with 5% CO_2_. Cells obtained from each donor were cultured at passages 2–6 and studied independently.

### Assessment of silymarin toxicity

Human primary chondrocytes (5000 cells/well in a 96-well tissue culture plate) were maintained in regular medium overnight and subsequently cultured in media containing 0, 12.5, 25, 50 and 100 μM silymarin (SMN, S0292, Sigma-Aldrich). After 24 h of culture, cell death and mitochondrial activity of chondrocytes were evaluated using the lactate dehydrogenase (LDH) assay kit (786-210, CytoScan™, G-biosciences, USA) and the CCK-8 reagent (TEN-CCK8, TOOLS Cell Counting CCK-8 kit, Tools, Taiwan), respectively. The cell morphology of SMN-treated chondrocytes was also recorded.

### Silymarin supplementation to IL-1β-stimulated chondrocytes

After treatment with 10 ng/mL IL-1β (579402, BioLegend, USA) for 24 h, the chondrocytes (40,000 cells/well in a 6-well tissue culture plate) were cultured in SMN-containing media (25 and 50 μM) for an additional 24 h. The LDH release and cell viability of the treated chondrocytes were determined again. In addition, the survival and senescence of the treated cells were evaluated using a live/dead double staining assay (R37601, LIVE/DEAD® Cell Imaging Kit, Thermo Fisher Scientific, USA) and β-galactosidase assay kit (β-gal, K320-250, Biovision, USA), respectively. Finally, glycosaminoglycan (GAG) production was detected in chondrocytes through toluidine blue staining under different treatments [18].

### mRNA expression of silymarin-treated chondrocytes

The treated chondrocytes were lysed for total RNA extraction (R2052 Direct-zol™ RNA MiniPrep Kit, Zymo, USA), and the RNA was reverse transcribed into cDNA (RR037A, PrimeScript™ RT Reagent Kit, TaKaRa, Japan). The gene expression in the cells was analysed using real-time polymerase chain reaction (real-time PCR; LightCycler 96®, Roche, Germany) with SYBR Green reagents (BIO-98005, SensiFAST™ SYBR NO-ROX Kit, Bioline Meridian, UK) for *inducible nitric oxide synthase* (*iNOS*), *IL*-*6*, *IL*-*8*, *IL*-*10*, *MMP*-*3*, *MMP*-*9*, *MMP*-*13*, *tissue inhibitor of metalloproteinase* (*TIMP*)-*1*, *aggrecan* (*AGCN*) and *sirtuin*-*1* (*Sirt1*, Table 1). Further, the Taqman system (ENZ-NUC 106-0200, AmpiGene™ qPCR Probe mix Hi-ROX, Enzo, USA) was used for the analysis of *IL*-*1β* (Hs01555410-m1), *TNF*-*α* (Hs01113624-g1), *collagen type II* (*COL2A1*, Hs00264051_m1) and *SOX9* (Hs01001343-g1) with a reference of the housekeeping gene glyceraldehyde 3-phosphate dehydrogenase (*GAPDH*) as a reference.

**Table 1:** The forward and reverse primers used for qRT-PCR analysis

Gene	Primer sequence
*iNOS*	Forward: 5′-CAGCGGGATGACTTTCCAA-3′
	Reverse: 5′-AGGCAAGATTTGGACCTGCA-3′
*IL-6*	Forward: 5′-GCCACTCACCTCTTCAGAACGA-3′
	Reverse: 5′-GGCAAGTCTCCTCATTGAATCC-3′
*IL-8*	Forward: 5′-ATCTGGCAACCCTAGTCTGCTA-3′
	Reverse: 5′-CTGTGAGGTAAGATGGTGGCTA-3
*IL-10*	Forward: 5′-CCCTGTGAAAACAAGAGCAAGG-3′
	Reverse: 5′-TCAGTTTCGTATCTTCATTGTC-3
*AGCN*	Forward: 5′-TCGAGGGTGTAGCGTGTAGAGA-3′
	Reverse: 5′-GCATCGAGGACAGCGAGG-3′
*TIMP-1*	Forward: 5′-TGGAAAACTGCAGGATGGACTC-3′
	Reverse: 5′-GTTTGCAGGGGATGGATAAACAG-3′
*MMP-3*	Forward: 5′-CGCATATGAAGTTACTAGCAAG-3
	Reverse: 5′-GCATCGATTTTCCTCACGGTTG-3
*MMP-9*	Forward: 5′-TTGACAGCGACAAGAAGTGG-3′
	Reverse: 5′′-TCACGTCGTCCTTATGCAAG-3′
*MMP-13*	Forward: 5′-TTGACAGCGACAAGAAGTGG-3′
	Reverse: 5′-TCACGTCGTCCTTATGCAAG-3′
*Sirt1*	Forward: 5′-ATGCTTCTAGGCGGACTATGACTT-3′
	Reverse: 5′-CACCTTCACCGTTCCAGTTT-3′
*GAPDH*	Forward: 5′-CACTCAGACCCCCACCACAC-3′
	Reverse: 5′-GATACATGACAAGGTGCGGCT-3′

### Quantifications of anabolic and catabolic cytokines

The anabolic and catabolic cytokines in the culture supernatants of the treated chondrocytes were determined using a relevant enzyme-linked immunosorbent assay (ELISA). The levels of IL-1β (437004, ELISA MAX™ Deluxe Set Human IL-1β, BioLegend), IL-6 (88-7066, IL-6 Human Uncoated ELISA Kit, Invitrogen, Thermo Fisher Scientific), IL-8 (BMS204/3, IL-8 Human ELISA Kit, Invitrogen, Thermo Fisher Scientific), IL-10 (88-7106, Invitrogen, Thermo Fisher Scientific), TNF-α (88-7346, TNF alpha Human Uncoated ELISA Kit, Invitrogen, Thermo Fisher Scientific), MMP-3 (444807, LEGEND MAX™ Human Total MMP-3 ELISA Kit, BioLegend), MMP-9 (440707, LEGEND MAX™ Human MMP-9 ELISA Kit with precoated plates, BioLegend), TIMP-1 (DY970, Human TIMP-1 DuoSet ELISA, R&D, USA) and TIMP-2 (DY971, Human TIMP-2 DuoSet ELISA, R&D) were quantified.

### Nicotinamide treatment of SMN-treated chondrocytes

To verify the role of *Sirt1* in ECM homeostasis in IL-1β-stimulated chondrocytes, 10 nM nicotinamide (NAM, 72340, Sigma-Aldrich), a *Sirt1* inhibitor, was added to the culture medium with 25 μM SMN [19]. After cultivation for 24 h, the mRNA levels of *AGCN*, *COL2A1*, *COL10*, *MMP*-*13*, *Sirt1* and *SOX9* were determined again through qPCR in the treated cells.

### Statistical analysis

The mean and standard error of the obtained data were calculated and analysed using analysis of variance and Tukey’s post hoc test for multiple group comparisons. At least six patients were included in one experiment, except the three patients included in the NAM study. A *p* value of < 0.05 was considered statistically significant.

## Results

### SMN in high doses impaired cell viability of articular chondrocytes

**Fig. 1:**
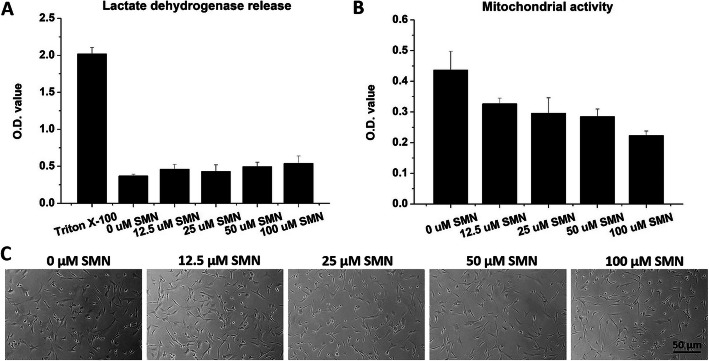
Cytotoxicity of SMN to articular chondrocytes. Human chondrocytes were incubated with the indicated concentrations of SMN for 24 h and then analysed. **a** SMN supplementation did not increase LDH release. **b** SMN additions reduced the mitochondrial activity in chondrocytes, and high-dose SMN (100 μM) further impaired cell viability. **c** No evidence of hypertrophic transformation or proliferation in SMN-treated chondrocytes was obtained

The LDH level and mitochondrial activity of the treated chondrocytes were determined. SMN supplementation (12.5–100 μM) had no effect on the LDH release (Fig. 1a) but significantly reduced the mitochondrial activity of the treated cells (*p* < 0.01 for the 12.5, 25 and 50 μM SMN groups and *p* < 0.001 for the 100 μM SMN group; Fig. 1b). In addition, chondrocytes treated with 100 μM SMN exhibited significantly lower cell viability than the other three SMN groups (*p* < 0.01). The treated chondrocytes did not exhibit a typical cobble-stone morphology (0 μM group), and SMN treatment caused a slightly cytoplasmic extension to chondrocytes (Fig. 1c). However, no hypertrophic differentiation or proliferation was found in the SMN-treated cells.

### Cell death, viability, GAG production, survival, and senescence after IL-1β stimulation and SMN supplementation

**Fig. 2:**
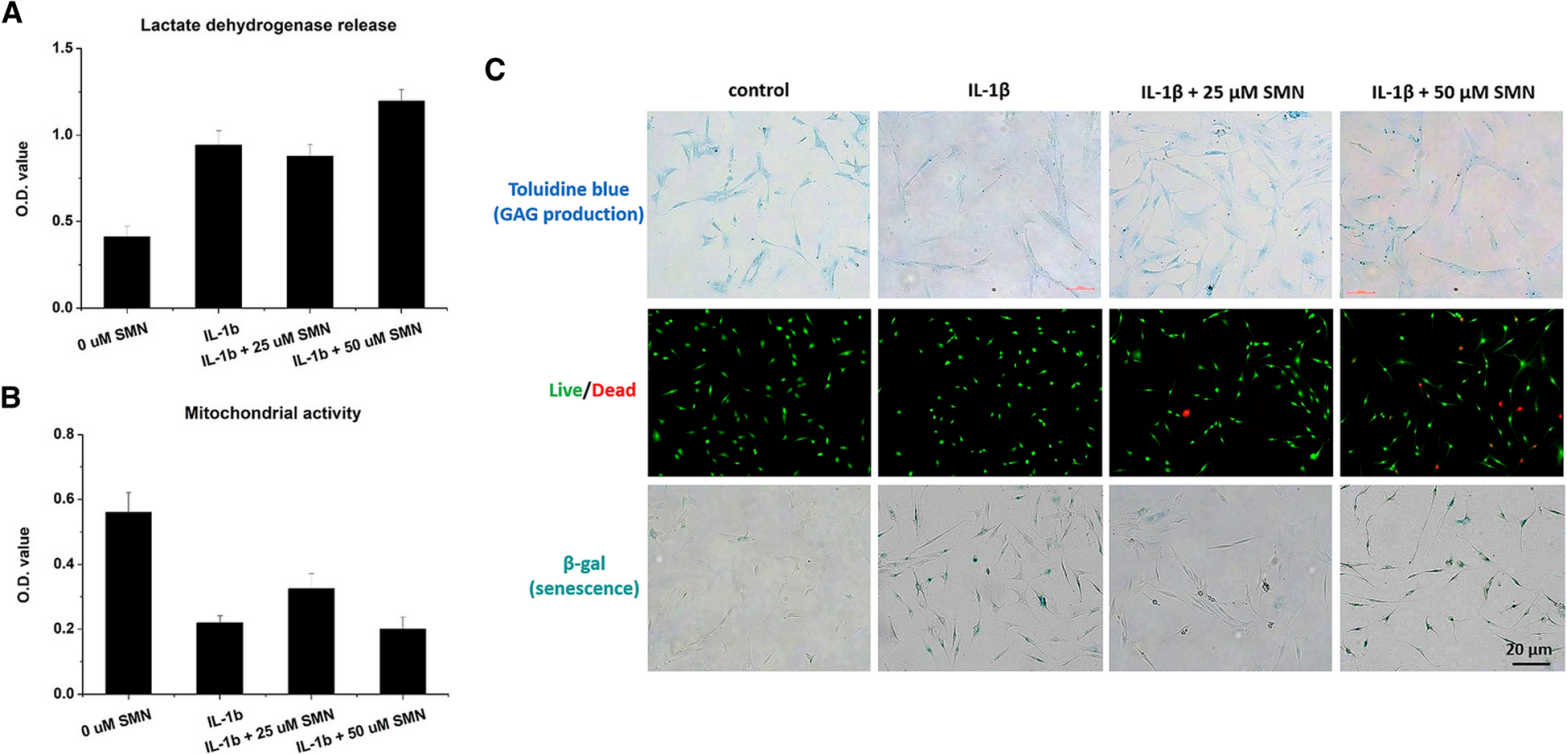
Treatment of IL-1β-stimulated chondrocytes with SMN. **a** Addition of 50 μM SMN further increased cell death (LDH release) of injured cells. **b** Addition of 25 μM SMN partially restored the viability of stimulated chondrocytes. **c** IL-1β stimulation impaired GAG production (toluidine blue staining), reduced the number of surviving cells (green fluorescence), and resulted in cell senescence (β-gal staining). Supplementation with 50 μM SMN had no substantial effects, whereas the addition of 25 μM SMN ameliorated GAG production, cell survival and β-gal expression in IL-1β-injured chondrocytes

IL-1β stimulation significantly increased cell death (LDH release, *p* < 0.01, Fig. 2a) and impaired mitochondrial activity (*p* < 0.01, Fig. 2b). Treatment of the IL-1β-stimulated chondrocytes with 25 μM SMN had no effect on cell death, whereas 50 μM SMN increased LDH release (*p* < 0.05). By contrast, no beneficial effects were observed on mitochondrial activity after treatment with 50 μM SMN, but treatment with 25 μM SMN improved cell viability during injury. IL-1β stimulation also impaired GAG production (toluidine blue staining, blue colour), reduced the number of surviving cells (green fluorescence) and resulted in cell senescence (β-gal staining, blue colour). Moreover, supplementation with 25 μM SMN ameliorated GAG production, cell survival and β-gal expression in IL-1β-injured chondrocytes (Fig. 2c).

### SMN modulated the mRNA expression of catabolic and anabolic cytokines

**Fig. 3:**
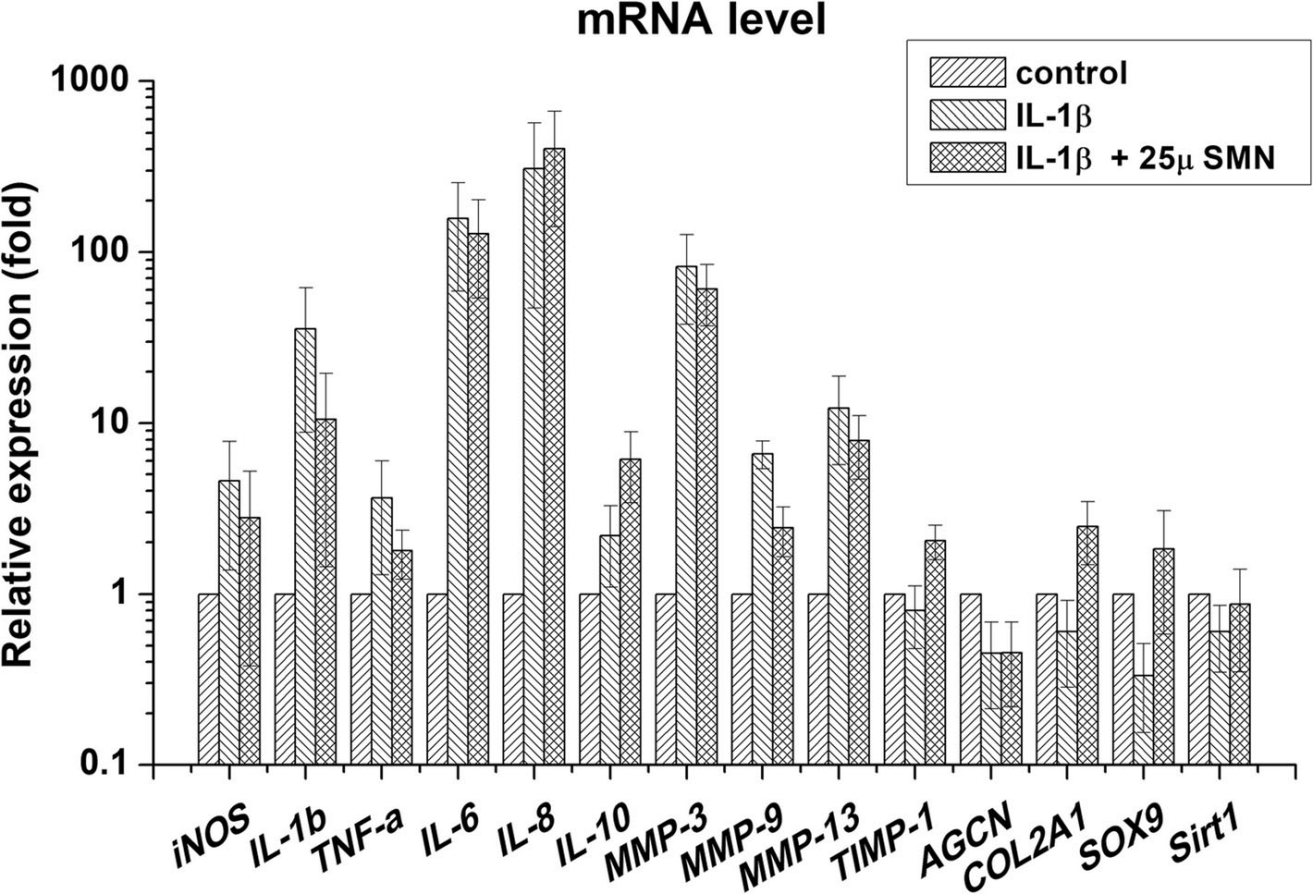
The mRNA expression profile of SMN-treated chondrocytes. Supplementation with 25 μM SMN significantly downregulated catabolic *iNOS*, *IL*-*1β*, *TNF*-*α*, *MMP*-*3*, *MMP*-*9* and *MMP*-*13* and upregulated anabolic *IL-10*, *TIMP*-*1* and *COL2A1* mRNA expression in IL-1β-stimulated chondrocytes. The chondrogenic phenotype genes *SOX9* and *Sirt1* were also restored. However, SMN did not cause substantial changes in the mRNA levels of *IL*-*6*, *IL*-*8* and *AGCN* in the injured cells

The results of real-time PCR showed that supplementation with 25 μM SMN significantly downregulated the IL-1β-stimulated expression of catabolic *iNOS* (*p* < 0.05), *IL*-*1β* (*p* < 0.05), *TNF*-*α* (*p* < 0.05), *MMP*-*3* (*p* < 0.05), *MMP*-*9* (*p* < 0.01), and *MMP*-*13* (*p* < 0.05) and upregulated anabolic *IL*-*10* (*p* < 0.05), *TIMP*-*1* (*p* < 0.05), and *COL2A1* (*p* < 0.01) in the chondrocytes. Moreover, the chondrocyte phenotype genes *SOX9* (*p* < 0.01) and *Sirt1* (*p* < 0.05) were restored. However, SMN did not cause substantial changes in the mRNA levels of *IL*-*6*, *IL*-*8*, and *AGCN* in the stimulated cells (Fig. 3).

### SMN modulated anabolic and catabolic cytokines

**Fig. 4:**
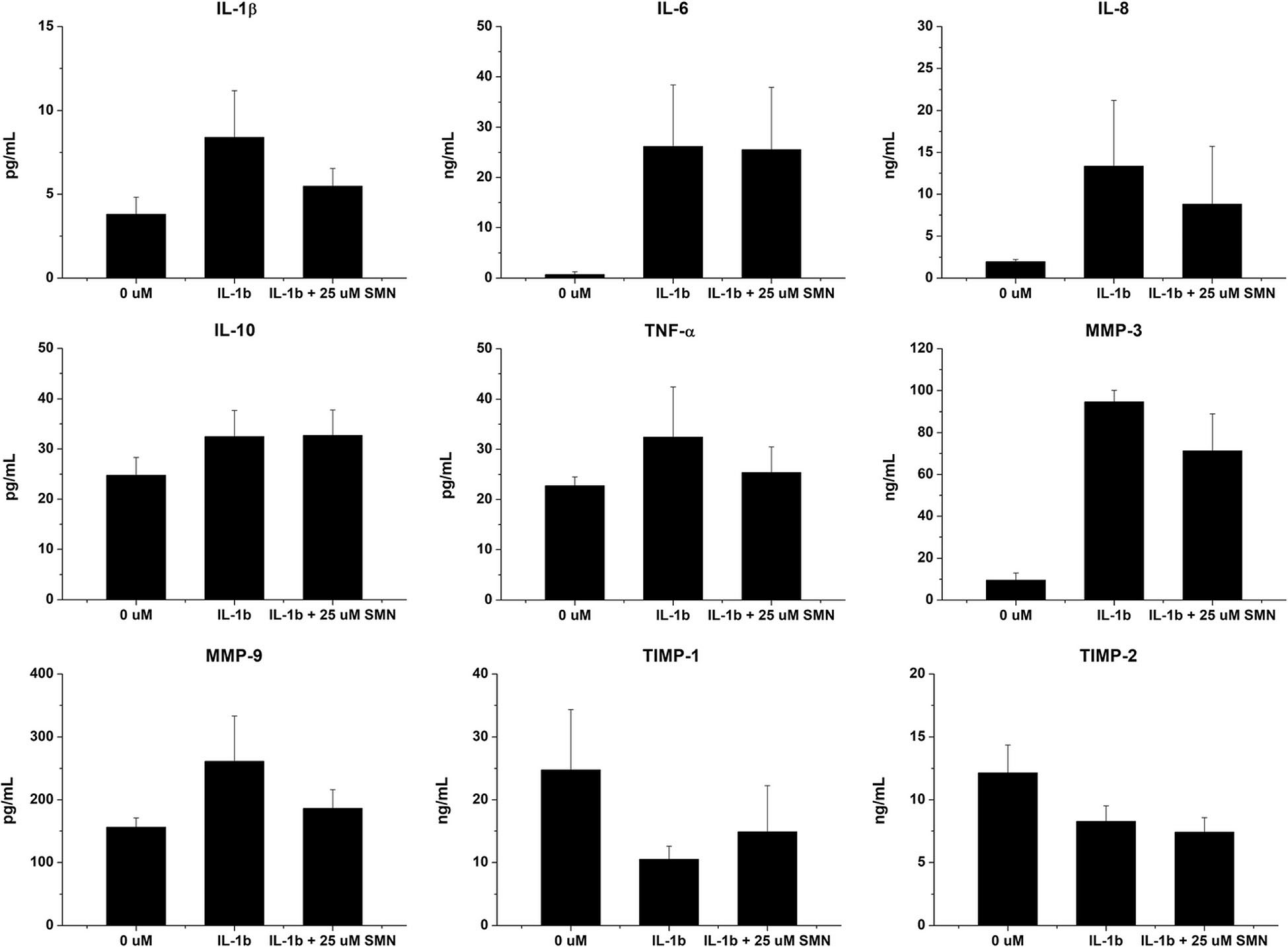
Modulation of anabolic and catabolic cytokines by SMN. ELISA assays revealed that treatment with 25 μM SMN reduced the production of catabolic cytokine-stimulated IL-1β, TNF-α, MMP-3 and MMP-9 and increased the production of TIMP-1 in the injured chondrocytes. However, the levels of IL-6, IL-8, IL-10 and TIMP-2 proteins remained unaltered upon SMN addition

SMN supplementation decreased IL-1β (*p* < 0.01), TNF-α (*p* < 0.05), MMP-3 (*p* < 0.01), MMP-9 (*p* < 0.01), but increased upregulated TIMP-1 (*p* < 0.05) productions in IL-1β-stimulated chondrocytes (Fig. 4). By contrast, the secretion of IL-6, IL-8, IL-10 and TIMP-2 was not altered upon SMN treatment.

### NAM downregulated *Sirt1* and *SOX9* in SMN-treated chondrocytes

**Fig. 5:**
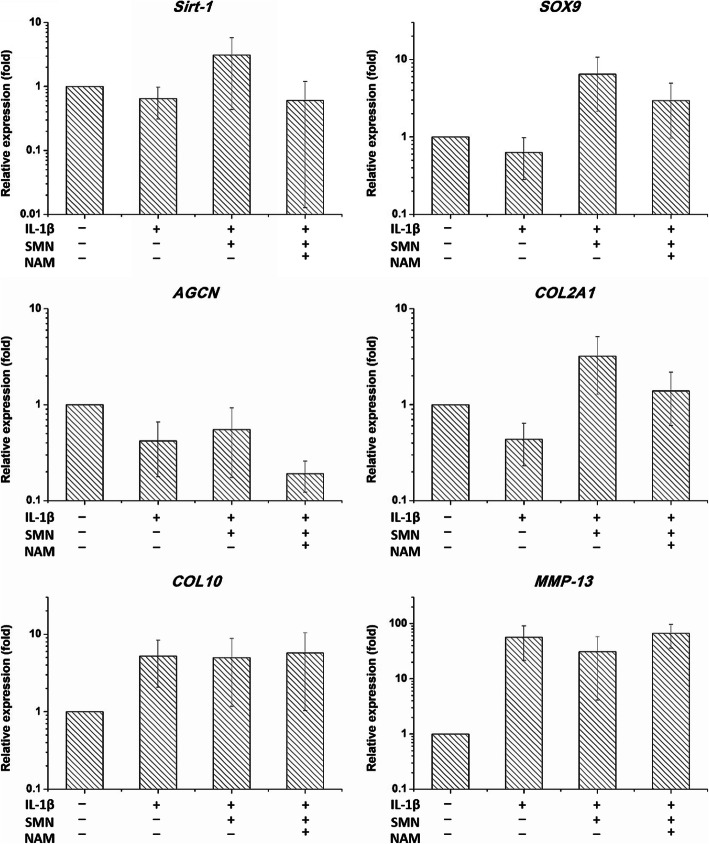
NAM attenuated the therapeutic effects of SMN, as evidenced by the downregulation of *Sirt*-*1*, *AGCN* and *COL2A1*

NAM additions downregulated *Sirt1* expression (*p* < 0.05) in IL-1β-stimulated chondrocytes even after SMN supplementation (Fig. 5). In spite of *SOX9* level was decreased, there was no significant difference between IL-1β + SMN and IL-1β + SMA + NAM groups. Similarly, the cartilaginous ECM genes *AGCN* (*p* < 0.05) and *COL2A1* (*p* < 0.05) were downregulated, whereas the hypertrophic genes *IL*-*10* and *MMP*-*13* were not changed in IL-1β + SMA + NAM group.

## Discussion

OA is one the most common degenerative joint diseases, and the application of phytochemicals is recommended to adjust ECM homeostasis during arthritis progression [20]. Because dietary nutraceuticals and antioxidants are generally not sufficiently effective for protection against OA development, direct administration of bioactive compounds into the knee joint capsule is a promising option [21, 22]. Pretreatment with SMN has been shown to alleviate TNF-α-stimulated inflammatory responses in chondrocytes, and SMN has also been used in combination with NASIDs to treat OA [10]. However, the therapeutic potential of SMN against catabolic cytokine stimulations is not fully elucidated. In particular, SMN pre- and posttreatment may exert different effects on OA chondrocytes. Therefore, this study demonstrated the effects of SMN posttreatment on IL-1β-stimulated human chondrocytes.

Although phytochemicals possess multiple bioactive functions, high doses have deteriorating effects on cells, especially the primary human OA chondrocytes [17, 23]. Dvořák et al. reported that SMN (10–100 μM) did not exert cytotoxicity (LDH release) toward primary human hepatocytes [24]. Similarly, Gharagozloo and Amirghofran demonstrated that SMN (50 and 100 μM) increased viability in Jurkat cells [25]. However, we found that SMN did not cause cell death (Fig. 1a), but rather reduced the mitochondrial activity (Fig. 1b) in primary chondrocytes. In addition, the responses of OA chondrocytes to external stimulation differ from those of healthy cells [23]. Our study demonstrated that 50 μM SMN supplementation did not cause cell death in unstimulated chondrocytes (Fig. 1a); however, cells with IL-1β-induced injury were more sensitive to SMN toxicity, and 50 μM SMN treatment resulted in cell death (Fig. 2a). Our findings revealed that the cytotoxic effects of SMN may be cell type-dependent. Although SMN is reported be cytoprotective, we found that LDH release and mitochondrial activity (Fig. 2b) were not fully restored in IL-1β-treated chondrocytes upon treatment with 25 μM SMN. Similarly, the GAG production, survival and senescence in chondrocytes were partially ameliorated (Fig. 2c).

Overexpression of catabolic cytokines such as IL-1β can trigger an inflammatory cascade, and the proinflammatory mediator-driven positive feedback loop contributes to the OA by promoting ECM destruction [26, 27]. Zheng et al. demonstrated that pretreatment with silibinin, the main active component of SMN, attenuated OA through inhibition of the expression of iNOS, TNF-α, NO, COX2 and prostaglandin E2 and cartilage ECM degradation in vitro [28]. Similarly, pretreatment with SMN regulated the mRNA expression of *IL*-*6*, *IL*-*8* and *MMP*-*1* which ameliorated the TNF-α-stimulated responses in chondrocytes [10]. Despite the downregulation of *iNOS*, *IL*-*1β* and *TNF*-*α* after SMN posttreatment, our results indicated that *IL*-*6* and *IL*-*8* expression was not restored in the stimulated chondrocytes (Fig. 3). Moreover, SMN posttreatment downregulated catabolic *MMP*-*3*, *MMP*-*9* and *MMP*-*13*; upregulated anabolic *IL*-*10* and *TIMP*-*1*; and enhanced the expression of ECM gene *COL2A1*. Similarly, the levels of chondrogenic phenotype genes *SOX9* and *Sirt1* were also restored. Because IL-6 is known to downregulate *COL2A1* and *AGCN* and IL-8 was found to induce hypertrophic transformation in chondrocytes [29, 30], our data suggest that SMN posttreatment may not affect ECM balance through the regulation of IL-6 and IL-8. In addition, *SOX9* and *Sirt1* were upregulated in our study, which is consistent with a previous study that demonstrated that the combination of SOX9 and SOX5/6 enhanced *COL2A1* transcription [31]. A similar study demonstrated that *COL2A1* expression is positively regulated by *Sirt1* through the chromatin-binding regions of the *COL2A1* promoter and enhancer, which implied that the disruption of *Sirt1* could accelerate OA progression during ageing [32, 33]. Taken together, SMN posttreatment may modulate ECM homeostasis through the regulation of *SOX9* and *Sirt1*.

Although SMN posttreatment upregulated *IL*-*10* mRNA level, IL-10 protein production was not further increased (Fig. 4). Ortved et al. found that *IL*-*10* expression was increased in IL-1β-stimulated chondrocytes, and overexpression of *IL*-*10* reduced the *IL*-*1β* levels but did not rescue GAG synthesis [34]. In addition, another study reported that IL-10 overexpression antagonised TNF-α-stimulated downregulation of *AGCN*, while the suppression of *COL2A1* was barely affected [35]. Although the SMN-treated chondrocytes exhibited upregulated *IL*-*10* levels and increased IL-10 secretion, the *AGCN* levels remained unchanged. Therefore, the correlation between SMN addition and IL-10 expression to ECM component production requires further investigation.

Regarding the role of *Sirt1* in cartilage homeostasis, several cartilage-specific genes such as *AGCN* and *COL2A1* are known to be modulated through *Sirt1*-mediated deacetylation of *SOX9* [36]. The *Sirt1* inhibitor NAM attenuated the therapeutic effects of SMN, as evidenced by the downregulation of anabolic genes such as *AGCN* and *COL2A1*, which revealed the correlations among *Sirt1*, *SOX9* and ECM components.

**Fig. 6:**
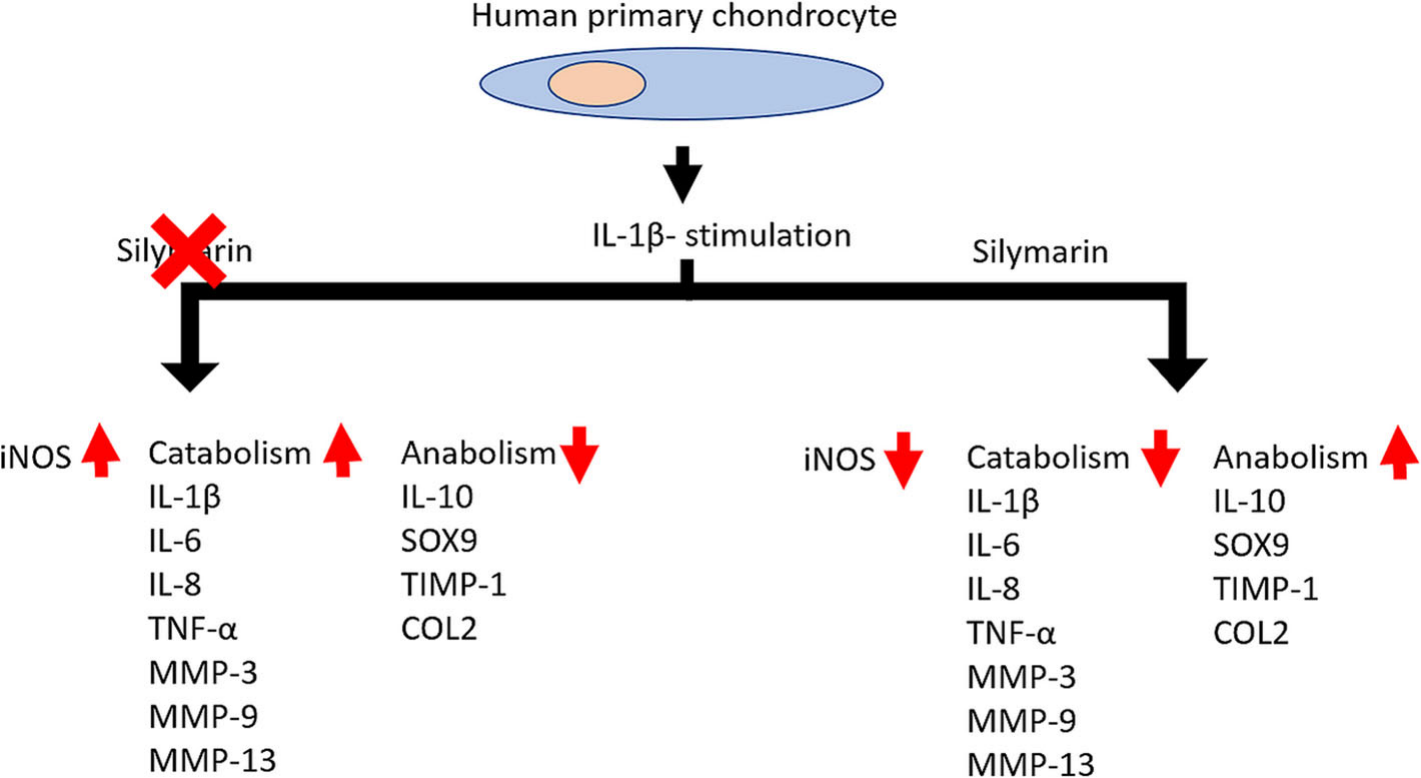
Regulation of SMN on anabolism and catabolism in inflammatory chondrocytes by SMN

In conclusion, high-dose SMN (100 μM) impaired the mitochondrial activity in unstimulated chondrocytes, and 50 μM SMN supplementation increased cell death in IL-1β-stimulated cells, suggesting that injured chondrocytes are more sensitive to SMN toxicity. Treatment with 25 μM SMN ameliorated cell senescence, downregulated catabolic mRNA expression and upregulated anabolic mRNA expression in IL-1β-stimulated chondrocytes. Furthermore, levels of IL-1β, MMP-3 and MMP-9 proteins were decreased, whereas that of TIMP-1 was increased. Although the *IL*-*6*, *IL*-*8* and *IL*-*10* mRNA levels as well as protein production were still high, the upregulation in *COL2A1* found in the current study revealed that SMN may maintain the functional phenotype of chondrocytes through the regulation of *Sirt1* and *SOX9* (Fig. 6). Therefore, SMN posttreatment may modulate catabolic cytokines to restore ECM homeostasis in OA chondrocytes.

## Data Availability

The necessary data were provided to support the assumption of this study (data will be made available on demand).

## Abbreviations

AGCN: Aggrecan
β-gal: β-galactosidase
COL2A1: Collagen type II alpha 1
ECM: Extracellular matrix
ELISA: Enzyme-linked immunosorbent assay
GAPDH: Glyceraldehyde 3-phosphate dehydrogenase
IL-1β: Interleukin-1 beta
IL-6: Interleukin-6
IL-8: Interleukin-8
IL-10: Interleukin-10
iNOS: Inducible nitric oxide synthase
LDH: Lactate dehydrogenase
MMP-3: Matrix metalloproteinase-3
MMP-9: Matrix metalloproteinase-9
MMP-13: Matrix metalloproteinase-13
NAM: Nicotinamide
NSAID: Nonsteroidal anti-inflammatory drug
OA: Osteoarthritis
PCR: Polymerase chain reaction
SMN: Silymarin
Sirt1: Sirtuin-1
TIMP-1: Tissue inhibitor of metalloproteinase-1
TIMP-2: Tissue inhibitor of metalloproteinase-2
TNF-α: Tumour necrosis factor-alpha
